# Computer Aided Detection System for Prediction of the Malaise during Hemodialysis

**DOI:** 10.1155/2016/8748156

**Published:** 2016-03-06

**Authors:** Sabina Tangaro, Annarita Fanizzi, Nicola Amoroso, Roberto Corciulo, Elena Garuccio, Loreto Gesualdo, Giuliana Loizzo, Deni Aldo Procaccini, Lucia Vernò, Roberto Bellotti

**Affiliations:** ^1^Istituto Nazionale di Fisica Nucleare, Sezione di Bari, Via Orabona 4, 70125 Bari, Italy; ^2^Dipartimento Interateneo di Fisica “M. Merlin”, Università degli studi di Bari “A. Moro”, Via Orabona 4, 70125 Bari, Italy; ^3^Nephrology, Dialysis and Transplantation Unit, University of Bari “A. Moro”, Piazza G. Cesare, 11 – Policlinico, 70124 Bari, Italy; ^4^Department of Physical Sciences, Earth and Environment, University of Siena, Strada Laterina 8, 53100 Siena, Italy

## Abstract

Monitoring of dialysis sessions is crucial as different stress factors can yield suffering or critical situations. Specialized personnel is usually required for the administration of this medical treatment; nevertheless, subjects whose clinical status can be considered stable require different monitoring strategies when compared with subjects with critical clinical conditions. In this case domiciliary treatment or monitoring can substantially improve the quality of life of patients undergoing dialysis. In this work, we present a* Computer Aided Detection* (CAD) system for the telemonitoring of patients' clinical parameters. The CAD was mainly designed to predict the insurgence of critical events; it consisted of two* Random Forest* (RF) classifiers: the first one (RF_1_) predicting the onset of any malaise one hour after the treatment start and the second one (RF_2_) again two hours later. The developed system shows an accurate classification performance in terms of both* sensitivity* and* specificity*. The* specificity* in the identification of nonsymptomatic sessions and the* sensitivity* in the identification of symptomatic sessions for RF_2_ are equal to 86.60% and 71.40%, respectively, thus suggesting the CAD as an effective tool to support expert nephrologists in telemonitoring the patients.

## 1. Introduction

Uremia is likely to occur when a person reaches the final stage of End Stage Renal Disease (ESRD) as in the presence of renal failure, urinary waste products, such as nitrogenous substances and in particular urea, accumulate in the blood. In these cases hemodialysis is the medical treatment replacing (but not restoring) the kidney function, thus allowing the extracorporeal removal of free water and waste products from the blood. For hemodynamically unstable patients hemodialysis is conducted in a dialysis outpatient facility. However, for hemodynamically stable patients the dialysis treatment can be also performed at home, for example, with the assistance of a trained person, the patient himself, or a family member.

ESRD is associated with premature mortality, decreased quality of life, and increased healthcare costs [1]. It is worthwhile to note that today dialysis patients represent only 1% to 2% of the population affected by chronic kidney disease [2]; nonetheless over the past three decades, the incidence of the ESRD has rapidly grown. Accordingly, this “silent epidemic” represents a huge burden on the national healthcare systems. In Europe, dialysis alone takes up about 2% of healthcare budgets with only a small proportion (<0.1%) of the population needing treatment [3]. These estimates are going to double its value within the next 5 years, not even considering other costs related to additional medical expenses, increased rate of morbidity, hospitalization, reduction in work capability, and life expectancy [4].

In particular, in Italy the annual hemodialysis cost per patient ranges between 35000 and 45000* euro*. ESRD patients represent only 0.083% of the Italian population; on the contrary the overall treatment cost per year is about 2.5% of the national healthcare budget (2,5 billion euro). As a consequence, there is a compelling necessity for appropriate healthcare policies and novel management solutions. With this aim, it is fundamental to encourage the development of protocols and methods granting the patients independence and decision-making power in order to mitigate the national healthcare burden. As an example, the Department of Health in London has shown how the dehospitalization policy of the hemodialysis patients can be a key solution: (i) yielding significant cost reductions (approximately 30–40%) and (ii) improving life quality [5]. A robust teleassistance/telemonitoring system, providing medical support to discharged patients at home, is a first fundamental step in this direction.

Home telemonitoring/assistance is a healthcare management approach based on remote monitoring of the patients. It consists of several sensor units collecting medical data, managing and sending it to the Call Center Server where trained staff perform data monitoring and support teleassistance services. In such a context, we designed and deployed a fully automated* Computer Aided Detection* (CAD) system, which is integrated into the telemonitoring platform. The CAD is designed to predict the insurgence of critical phenomena such as hypotension during each hemodialysis session. The CAD main result is a warning signal that suggests qualified personnel to directly inspect primary clinical parameters, eventually to provide adequate intervention.

During a dialysis session, in fact, several conditions related to intradialytic hypotension such as nausea, muscle cramps, and dizziness may occur. Intradialytic hypotension can be defined as a reduction of systolic blood pressure exceeding 20 mmHg or as a reduction of the average pressure of 10 mmHg [6]. It attains from 5% to 30% of hemodialysis sessions [7], with this variability depending mostly on the heterogeneity of clinical conditions, and it can occur at least one time for 75% of patients [8]. Occurrences higher than 50% have also been reported [9]. Dialysis hypotension is caused by a decrease of blood volume due to the imbalance between the ultrafiltration rate and the plasma refilling rate [10]. Furthermore in case of hypotension, it is mostly impossible to reach the dry weight leading to a state of chronic overhydration [11–14].

The occurrence of hypotensive phenomena could be lessened by performing hemodialysis at home, but in this case the forecast of malaise conditions is of paramount importance in order to grant patients the same care standards they would benefit from in a dialysis outpatient facility. In the last 20 years the prevention of intradialytic hypotension phenomena and the related causes have been deeply investigated [10], in particular exploring the use of innovative noninvasive techniques for the patient monitoring. These techniques are mostly based on the monitoring of the Relative Blood Volume (RBV), which varies during the hemodialysis session and should maintain an adequate intravascular value [15–17]. A large amount of clinical and biochemical data is available but difficult to interpret because of noise acquisition or data heterogeneity, just to mention a few possible causes. In principle, supervised machine learning techniques could be able to tackle these difficulties and accurately model the occurrence of malaise during hemodialysis [18–21]. However, the methods proposed in the literature elaborate numerous variables known or supposed to influence the risk of hypotensive phenomena according to clinical experience, but hardly detectable for patients in session at home.

On the contrary, in this work we present here a supervised CAD based on primary clinical parameters that patients can easily collect at home. In particular, the CAD is based on the monitoring of systolic and diastolic blood pressure, heart rate, and weight loss. This choice is motivated by expert nephrologists keeping in mind the goal which is not secondary to prevent the occurrence of potentially high risk situations. For example, both high and low levels of systolic and diastolic blood pressure are associated with organ damage and serious vascular complications, such as cerebral infarction and cardiac and mesenteric ischemia [22, 23]; therefore these parameters must be monitored to effectively assist patients undergoing home hemodialysis. The CAD was developed on data collected in a sample of patients characterized by a stable clinical picture suitable for home hemodialysis. A Random Forest [24] classifier was adopted for both its robustness to failure and its computational efficiency. To improve the monitoring quality the CAD was designed to predict the onset of any malaise already one hour after the treatment start and again after 120 minutes.

## 2. Materials

Data used in preparation of this paper were appositely collected as part of an experimental study from May to October 2014 on a total of 10 patients (7 men and 3 women) undergoing hemodialysis at the Nephrology Department of Bari University. Patients selected for home dialysis presented a stable clinical picture, that is, with their clinical parameters not showing significant variations during the sessions.

The treatments were performed three times a week, with each treatment lasting up to 240 minutes. We acquired data at several times *Tα* from the session start (*T*0) up to the end with an interval of 30 minutes (*T*1, *T*2, *T*3, *T*4, *T*5, *T*6, *T*7, and *T*8). We monitored and recorded the clinical parameters of interest as follows:

- systolic blood pressure (SBP),
- diastolic blood pressure (DBP),
- heart rate (HR),
- weight.

In clinical practice, physicians set the weight that the patient should reach at the end of the hemodialysis session and, consequently, the weight loss per hour on the machine.  Table 1 lists the parameters basic information measured for the ten patients.

Accordingly, the data included an overall amount of 200 sessions, each of them including the parameters described above. 8.8% of the sessions (17 sessions) were characterized by malaise (nausea, muscle cramps, and dizziness), and, in particular, for 14 sessions the symptoms occurred in the last hour of treatment. The statistical evidences emerging during the study are shown and discussed in the following.

### 2.1. Statistical Data Analysis

Dividing the observations in two samples of patients, presenting or not hypotensive phenomena, we also performed the hypothesis tests on the average to eventually outline the presence of significant differences. In fact, statistical Student's *t*-test for independent samples showed that after 30 minutes (*T*1) from the beginning of the session, after 90 minutes (*T*3), and then until the end, the average values of SBP and DBP of the sample of symptomatic sessions were significantly different from those found in the sample of nonsymptomatic sessions (Table 2). This observation suggested a possible correlation of SBP and DBP with the malaise onset.

Also, we found a significant difference between the average weight loss measured at *T*7 and *T*6, which is indeed the time interval presenting the highest number of hypotensive phenomena (Table 3).

Throughout the hemodialysis treatment it was not possible to detect any significant difference between the mean values of HR. However, it should be emphasized that the heart rate is a parameter characterized by a more dynamic trend than blood pressure, because it rapidly responds to metabolic demands of the individual. As a consequence a 30-minute time window could not probably be an optimal choice to detect symptomatic changes, although one of the defensive mechanisms of the cardiovascular system is sinus (or not) tachycardia, when the blood pressure is dropping.

Interestingly, we found a linear relation between the recorded parameters in each of the two groups. In particular, statistical Student's *t*-test on the Bravais-Pearson correlation coefficient showed a significant correlation between SBP, DBP, HR, and the patient's weight recorded at the same time (Table 4) for patients with no symptoms, except for *T*0 and *T*1 cases. On the contrary, in presence of symptoms, it is worth noting that such a linear relation was not significant (Table 5).

In line with the CAD purpose, we included two other variables potentially useful in predicting the onset of malaise. The first variable we added was the interdialytic weight gain. Indeed, one of the possible causes of malaise during the session could be likely attributed to an excessive initial weight yielding a too fast removal of fluids in relation to the time set for the dialysis [25, 26]. The interdialytic weight gain was measured as the variation in percentage between the weight at the beginning of the session (*T*0) and the final weight of the previous session (*T*8′) divided by the estimated dry weight:

(1) $$\begin{array}{c} \Delta_{peso}=\frac{Weight\left(T0\right)-Weight\left(T8^{\prime }\right)}{Weight_{Dry}}. \end{array}$$

The second variable we included was calculated by the values of SBP at the beginning of the treatment. We observed how acute hypotensive phenomena were a nonsymptomatic event frequently occurring when the patient SBP at the beginning of session resulted higher than 100 mmHg, then dropping below 90 mmHg during the treatment [27]. Accordingly, for each session *i* and for each time detection *j* after the initial time, we defined the normalized* critical indicators *PP_*ij*_ ranging between 0 (minimum criticality) and 1 (maximum criticality). Whenever a patient showed an initial SBP value higher than 100 mmHg the relative PP_*ij*_ values were computed according to the formula:

(2) PPij=1,SBPTij≤90 mmHg,PPij=1−SBPTij−90100−90,90 mmHg<SBPTij<100 mmHg,PPij=0,SBPTij≥100 mmHg.

On the contrary, for patients having an initial SBP < 100 mmHg the critical PP_*ij*_ value was set to zero.

## 3. Methods

**Figure 1:**
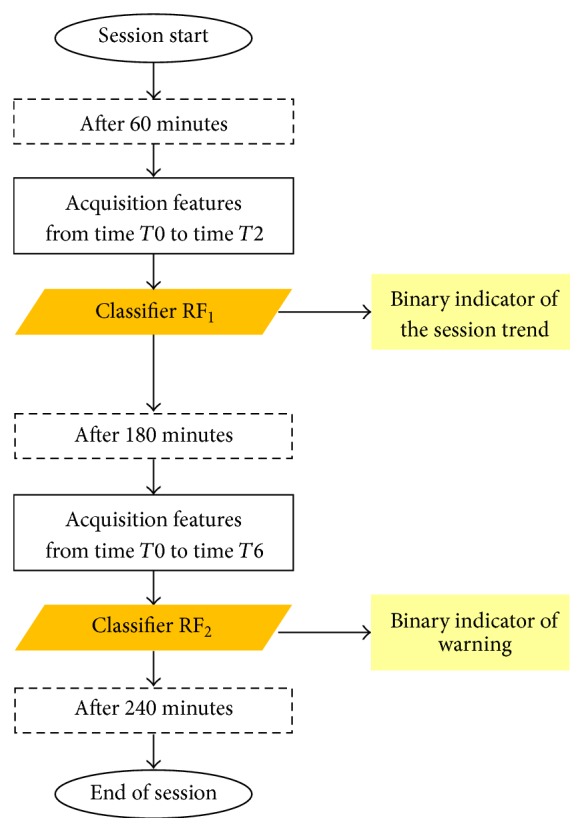
Timeline flowchart of the monitored hemodialysis session. The patients' monitoring yielding the measurement of features after 60 and 180 minutes is emphasized; in particular two Random Forests (RF) are used to detect the session trend and eventually a malaise warning.

We observed that *T*1 values of SBP, DBP, and HR are significantly associated with a malaise condition. Besides, with an incoming crisis phenomenon, these values did not show any correlation with the patient weight. Hence, we concluded that a one-hour monitoring window could be sufficient to provide a first robust alert. This warning signal was considered as a preliminary measurement of the session trend; however it was inevitably affected by important fluctuations (Figure 1) caused by the session start. Accordingly we designed the CAD to provide a second warning one hour before the end of the treatment. This design decision was also motivated from the fact that most hypotensive phenomena occurred during this particular time interval.

The recorded parameters and the previously mentioned auxiliary variables were included as input features of a* Random Forest* (RF) classifier. The RF is a well-known* ensemble* classification method consisting of a randomized set of stochastic tree classifiers. Each tree develops an independent classification for the same example; then the final classification is obtained by majority voting. The RF is characterized by a generalization error that converges as the number of trees in the forest becomes larger [28, 29]. The use of a random feature selection for the training of each tree grants RF an outstanding robustness and classification performance higher than or comparable to those obtained by other classifiers, such as AdaBoost, but more robust with respect to noise [30, 31]. Moreover, it allows managing a large number of features, maintaining its efficiency even in case of missing data and in the presence of outliers. Thus it is a classification algorithm suitable for clinical data analyses [32, 33].

The first RF_1_ classifier calculated a first risk likelihood for the hemodialysis session only considering the values of SBP, DBP, HR, and weight, measured at the beginning of the session and after 60 minutes. It also considered the values of weight loss per hour (the weight that should be reached at the end of the treatment). Additional features were the interdialytic weight gain and the critical indicators of PP at 30 and 60 minutes. A label vector, indicative of a symptomatic session (value 0) or nonsymptomatic session (value 1), was used for training. In this first phase, the binary vector considered all of the 17 sessions characterized by malaise. Analogously, the second RF_2_ classifier used the same clinical parameters measured at the beginning of the session and after 180 minutes. For this second model the label vector used for training did not consider the crises occurring during the past three hours of the treatment. The scores generated by the second classifier were averaged with the first ones in order to increase the power and strength of the final prediction.

As the dataset mainly consisted of nonsymptomatic sessions (183/200), it was necessary to balance the groups for the two classifiers' training. Undersampling the dataset would have yielded a very limited sample training (~30 observations). Thus, to solve this problem we preferred an oversampling strategy. We generated new examples for the minority class to balance the two-class problem with the addition of random noise to the observed features as suggested in [34].

Specifically, we simulated 166 new sessions on the basis of the statistical constraints previously emerging. We verified the normal distribution hypothesis for all the detected parameters and for each acquisition time *Tα*, at 1% significance level, by means of the nonparametric Kolmogorov-Smirnov test. Then we randomly generated the parameters for the new sessions, adding a white Gaussian noise. Finally, we combined these variables in order to reproduce the correlation between the parameters. In fact, some parameters are evidently correlated to each other and, for example, we found a significant correlation (1% significance) also between the following:

- weight to be reached at the end of the treatment and weight at the beginning of the treatment (Bravais-Pearson coefficient of 0.99),
- weight loss and absolute weight loss after one hour (coefficient of Bravais-Pearson equal to 0.99),
- interdialytic weight gain and weight loss per hour (Bravais-Pearson coefficient of 0.48),
- weight at the beginning of session and weight of any other detection (Bravais-Pearson coefficient of 0.99 for each detection time),
- SBP and DBP of any detection (Table 6).

Moreover, given the significance of the normal distribution hypothesis for all the detected parameters, first we randomly generated the parameters for the new sessions, adding a white Gaussian noise, and then we reproduced the correlation between the parameters, by combining these variables.

In general, given two variables *u* and *v* with mean equal to zero and variance equal to one and with Bravais-Pearson correlation coefficient *ρ*, the variables

(3) u′=u1−ρ2+vρ,v′=v

still have an average of zero and a variance of one, but their correlation coefficient is equal to *ρ*.

Accordingly, we assumed that *σ*_*x*_ and *σ*_*y*_ were the standard deviations and *μ*_*x*_ and *μ*_*y*_ the averages found in the symptomatic sessions of two parameters *x* and *y*. Once the variables *u*′ and *v*′, correlated by a coefficient *ρ*_*xy*_, were generated, we defined the two new variables *x*′ and *y*′ as

(4) x′=μx+kσxu′,y′=μy+kσyv′

with standard deviation *kσ*_*x*_ and *kσ*_*y*_, averages *μ*_*x*_ and *μ*_*y*_, and the same correlation *ρ*_*xy*_ of the two parameters *x* and *y* of the examined sample. The coefficient *k* was the proportionality factor of the standard deviation in the Gaussian noise. The validation of the model was performed with a* leave-one-out* cross-validation technique [35]. The decision to adopt this approach is derived from the small number of critical events in the class of interest.

**Figure 2:**
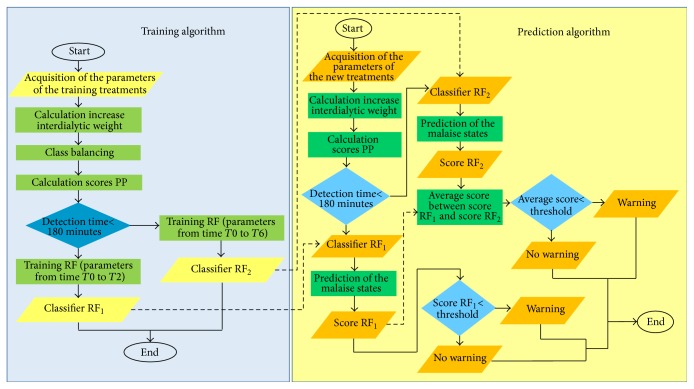
The figure shows a comprehensive overview of the training (blue box) and the prediction algorithm (yellow box).

In summary, as shown in Figure 2, the system was developed in two phases, the training phase (blue box) and the prediction phase (yellow box): two distinct predictions were available for the test session, on the basis of the two previously trained classifiers. The described classification system was implemented by means of a MATLAB statistical software.

The classification performance of the two classifiers was evaluated in terms of* accuracy*,* sensitivity,* and* specificity* and by means of the* Area Under the Curve* (AUC). By varying the threshold on the classification output, different values of sensitivity and specificity and, therefore, of accuracy were defined. The* Receiver Operating Characteristic* (ROC) curve summarized the classification performance along the entire range of the possible threshold values. The AUC of the ROC was used to express the “predictive power” of the classifier [36].

## 4. Results

**Figure 3:**
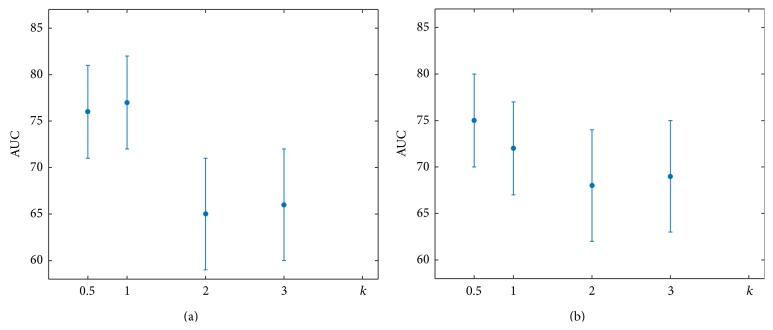
The AUC comparison of both classifiers for different values of the proportionality factor *k* of the standard deviation in the Gaussian noise.

The stability and the performance of the two classifiers were evaluated in terms of the AUC, by varying the constant *k* in the Gaussian noise formula. The classification performance was robust for both classifiers as shown in Figure 3. Besides, AUC results were not different (*z* test [37] significant at level 1%). The following results were obtained by simulating the sessions with events, setting the proportionality factor *k* equal to 1.

**Figure 4:**
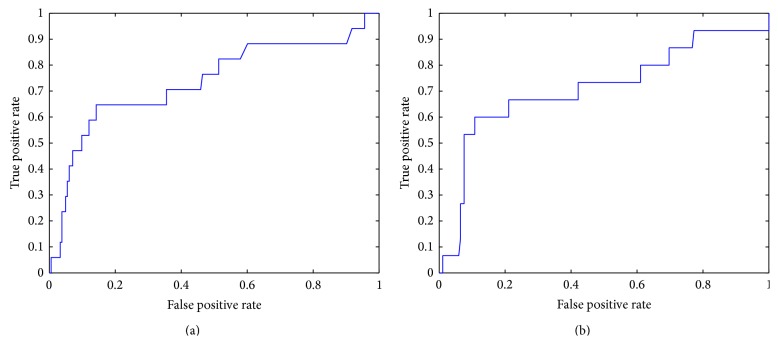
ROC curve of the first (a) and the second (b) classifier.

The classifier trained on the parameters of the first hour of hemodialysis and the other trained to predict the crisis in the last hour of treatment were moderately accurate [38]. In particular, the AUC of the first classifier was equal to 0.76 with an error of 0.05 (Figure 4(a)); the AUC of the second was equal to 0.73 with an error of 0.05 (Figure 4(b)). Using* Youden test* [39], it was possible to identify the best cutoff, that is, the optimal threshold that maximizes the predictive power of a classifier, as the difference between true positives (*sensitivity*) and false positives (1 − *specificity*).

The best cutoff maximizing the difference between true positives and false positives for the first classifier was equal to 0.42, associated with an overall* accuracy* of 86.50%. The best cutoff for the second classifier was 0.37 associated with an overall* accuracy* of 85.00% (Table 7). It is worth noting that the sensibility of the second was significantly higher, thus resulting in a more desirable situation in which more cases of malaise were detected.

It should be emphasized that the training of the two classifiers may be impaired by the presence of hypotensive nonsymptomatic phenomena. In fact, in these cases, important variations of the clinical parameters do not match the onset of malaise. Both classifiers had a high predictive power on sessions without critical events, respectively, accounting for 96.40% and 97.60%, but low predictive power on sessions with crisis events, respectively, equal to 34.40% and 27.80%.

## 5. Conclusions and Discussions

ESRD patients which regularly undergo hemodialysis may present malaise during the treatment eventually inducing severe risk situations. This is why the development of methodologies and analysis tools preventing the insurgence of critical events during the dialysis treatments deserves dedicated effort. Moreover, the successful development of strategies allowing the patients' dehospitalization would have a significant impact on both the quality of life of patients and the economic burden national healthcare systems have to face nowadays.

In this regard, many previous studies have investigated the adoption of noninvasive devices to monitor and to modify various parameters associated with the pathogenesis of malaise. Essentially, current research and clinical practice devices involve a complex instrumentation intended to monitor the volume of blood. However, there seems to be no direct control over some easily detectable clinical parameters, such as BP and HR, strongly associated with the occurrence of critical events. The pathogenic mechanism underlying the intradialytic hypotension (a progressive hypovolemia associated with an inadequate neuroadrenergic cardiovascular response) occurs progressively over time. It could be possible to hypothesize that the primary clinical parameters (blood pressure, body weight, and heart rate), along with the ultrafiltration volume measurement provided on the dialysis screen, can result in a robust base of knowledge for the design of an automatic prediction system for hypotensive phenomena.

Accordingly, we propose a CAD system that, starting from such easily detectable clinical parameters, is able to forecast the risk of hypotensive phenomena during a hemodialysis session. The model presented here is an innovative tool for remote monitoring and support for home dialysis patients. We demonstrated how this tool can reliably monitor the patients and effectively forecast the insurgence of hypotensive phenomena. The data heterogeneity and the class imbalance made could significantly lower the performance of whatever classification model; nonetheless we found our results more than satisfactory. It should also be noted that the problem is intrinsically imbalanced as the CAD is designed for hemodynamically stable patients, thus resulting in subjects with poor propensity to malaise. Further studies on independent datasets should be led in the future.

The performed analyses confirmed the relevance of some clinical parameters, such as the SBP and DBP levels, the HR and the body weight, and the dry weight and weight loss per hour to predict the insurgence of hypotensive phenomena. In particular we found that early alterations in SBP and DBP, probably due to the physical stress, can yield a crisis event. Interestingly, when these parameters did not stabilize within the first 90 minutes, we frequently observed a crisis event. In particular, our results would suggest that linear correlations among these variables and the weight could be good predictors.

On the basis of such information, we designed the CAD system, using a well-known* ensemble* method named* Random Forest*. The system provided two different warnings during a session. A patient undergoing hemodialysis eventually received a first signal alert after one hour of treatment, as a preliminary measure of the dialysis session. This warning was used to notify qualified personnel of the evidence of an abnormal condition (e.g., when patients needed to be stabilized). Since most of the hypotensive phenomena occurred 30 minutes before the session ends, a second signal was provided one hour before the end of the treatment.

The results of this first experimental study for hemodialysis telemonitoring were encouraging. Indeed, the performance of the two classifiers, evaluated by* leave-one-out cross-validation* technique, resulted moderately accurate, with both an AUC equal to 0.76 ± 0.05 and 0.73 ± 0.05 and an overall* accuracy* of 86.50% and 85.00%, respectively. It is worth noting that the first classifier was able to reach a higher AUC value; nevertheless the sensibility of the second one was significantly higher, thus resulting in a more desirable situation where fewer cases of malaise were not detected. The misclassified sessions resulted the same for both models, thus suggesting further investigation of these events in order to understand if misclassification is yielded, for example, by missing information (other clinical features could be considered in case) or they represented cases of asymptomatic malaise. Thus, about two-thirds of the alarm signals were not followed by any malaise event, probably because they were hypotensive nonsymptomatic phenomena.

The performance and strength of the designed system can be improved by increasing the dataset especially in case of hemodialysis sessions with new events of malaise, but also by the identification of a third class represented by nonsymptomatic hypotensive phenomena. In the present study, nonsymptomatic hypotensive phenomena were considered as a subsample of latent asymptomatic sessions, but it is reasonable to assume that they may have introduced distortions in the training phase of the designed model. Moreover, the identification of this other class should be useful to early diagnose and to reduce these kinds of phenomena, harmful for the patients.

Future developments of this study may relate the definition of a forecasting model specific for each patient, trained on his own historical data or on the data of patients with similar physical and clinical characteristics. The trained model, when properly integrated on telemonitoring and teleassistance platform, allows the patients to be monitored in real time, in order to favor the timely remote intervention of doctors in prevision of malaise.

## Figures and Tables

**Table 1:** Basic information about the parameters measured for the ten patients.

Subjects	Age	Sex	Critical events	Average weight (kg)	Average SBP (mmHg)	Average DBPe (mmHg)	Average HR (bpm)
Sbj 1	66	M	0	80.6 ± 3.3	143.2 ± 8.2	70.4 ± 4.2	68.6 ± 3.5
Sbj 2	65	M	3	93.7 ± 3.8	118.2 ± 8.3	64.6 ± 3.7	58.1 ± 4.1
Sbj 3	65	M	5	72.1 ± 3.1	111.9 ± 10.8	54.5 ± 6.2	78.0 ± 4.5
Sbj 4	65	M	6	80.0 ± 3.3	117.4 ± 9.3	65.0 ± 5.8	67.4 ± 5.3
Sbj 5	37	M	2	60.2 ± 3.0	109.5 ± 7.3	71.9 ± 4.9	66.5 ± 4.6
Sbj 6	47	M	0	47.9 ± 2.4	130.5 ± 4.7	74.5 ± 3.4	72.8 ± 3.3
Sbj 7	52	M	0	82.7 ± 5.2	116.4 ± 6.5	75.9 ± 4.3	67.2 ± 4.2
Sbj 8	67	F	0	62.0 ± 2.9	131.3 ± 7.9	82.5 ± 4.0	69.8 ± 3.1
Sbj 9	69	F	1	61.8 ± 2.6	151.6 ± 8.7	84.2 ± 5.6	69.3 ± 4.6
Sbj 10	68	F	0	92.0 ± 3.7	124.3 ± 7.9	59.5 ± 4.1	70.3 ± 3.8

**Table 2:** Differences between the average values of systolic blood pressure (SBP) and diastolic blood pressure (DBP) registered in the two classes of sessions, for each time *t* of detection.

	Parameters	
	SBP (mmHg)	DBP (mmHg)
*T*0	−7.86	−4.43
*T*1	−9.34^*∗*^	−5.92^*∗∗*^
*T*2	−7.33	−4.68
*T*3	−12.53^*∗∗*^	−6.01^*∗*^
*T*4	−13.80^*∗∗*^	−7.22^*∗∗*^
*T*5	−16.69^*∗∗*^	−8.80^*∗∗*^
*T*6	−16.43^*∗∗*^	−8.21^*∗∗*^
*T*7	−22.70^*∗*^	−12.97^*∗*^
*T*8	−21.52^*∗∗*^	−7.39^*∗*^

^*∗*^ *p* value < 0.05; ^*∗∗*^ *p* value < 0.01.

**Table 3:** Differences between the average values of weight loss recorded in the two classes of sessions, for each time *t* of the detection.

Parameter	*T*0	*T*1	*T*2	*T*3	*T*4	*T*5	*T*6	*T*7	*T*8

Weight loss (Kg)	—	0.10	0.09	0.14	0.01	0.06	0.05	0.14^*∗*^	−0.78

^*∗*^ *p* value < 0.05.

**Table 4:** Bravais-Pearson correlation coefficients between weight and systolic blood pressure (SBP), diastolic blood pressure (DBP), and heart rate (HR) recorded during the asymptomatic sessions.

	Weight (Kg) versus SBP (mmHg)	Weight (Kg) versus DBP (mmHg)	Weight (Kg) versus HR (bpm)
*T*0	0.04	−0.49^*∗∗*^	−0.25^*∗∗*^
*T*1	−0.11	−0.45^*∗∗*^	−0.29^*∗∗*^
*T*2	−0.15^*∗*^	−0.50^*∗∗*^	−0.25^*∗∗*^
*T*3	−0.15^*∗*^	−0.55^*∗∗*^	−0.25^*∗∗*^
*T*4	−0.16^*∗*^	−0.49^*∗∗*^	−0.23^*∗∗*^
*T*5	−0.22^*∗∗*^	−0.45^*∗∗*^	−0.29^*∗∗*^
*T*6	−0.28^*∗∗*^	−0.47^*∗∗*^	−0.35^*∗∗*^
*T*7	−0.33^*∗∗*^	−0.46^*∗∗*^	−0.33^*∗∗*^
*T*8	−0.30^*∗∗*^	−0.64^*∗∗*^	−0.30^*∗∗*^

^*∗*^ *p* value < 0.05; ^*∗∗*^ *p* value < 0.01.

**Table 5:** Bravais-Pearson correlation coefficients between the weight and systolic blood pressure (SBP), diastolic blood pressure (DBP), and heart rate (HR) recorded during the symptomatic sessions.

	Weight (Kg) versus SBP (mmHg)	Weight (Kg) versus DBP (mmHg)	Weight (Kg) versus HR (bpm)
*T*0	−0.59^*∗∗*^	−0.63^*∗∗*^	−0.53^*∗∗*^
*T*1	−0.22	−0.35	−0.46
*T*2	−0.22	0.13	−0.47
*T*3	−0.13	−0.04	−0.43
*T*4	−0.18	−0.15	−0.34
*T*5	−0.06	−0.19	−0.47
*T*6	−0.31	−0.16	−0.54^*∗*^
*T*7	0.23	0.06	−0.29
*T*8	−0.15	−0.16	−0.34

^*∗*^ *p* value < 0.05; ^*∗∗*^ *p* value < 0.01.

**Table 6:** Bravais-Pearson correlation coefficients between systolic blood pressure (mmHg) and diastolic blood pressure (mmHg) recorded during symptomatic dialysis sessions.

*T*0	*T*1	*T*2	*T*3	*T*4	*T*5	*T*6	*T*7	*T*8
0.76^*∗∗*^	0.76^*∗∗*^	0.83^*∗∗*^	0.83^*∗∗*^	0.84^*∗∗*^	0.80^*∗∗*^	0.85^*∗∗*^	0.57^*∗∗*^	0.70^*∗∗*^

^*∗∗*^ *p* value < 0.01.

**Table 7:** Performance indicators of the two classifiers.

	Classifier	
	RF_1_	RF_2_
AUC ± SE	0.76 ± 0.05	0.73 ± 0.05
Accuracy	86.50%	85.00%
Specificity	88.50%	86.00%
Sensitivity	64.70%	71.40%
